# Immunogenicity of COVID‐19 mRNA vaccines in hemodialysis patients: Systematic review and meta‐analysis

**DOI:** 10.1002/hsr2.874

**Published:** 2022-10-03

**Authors:** Shahab Falahi, Hojjat Sayyadi, Azra Kenarkoohi

**Affiliations:** ^1^ Zoonotic Diseases Research Center Ilam University of Medical Sciences Ilam Iran; ^2^ Department of Biostatistics, Faculty of Health Ilam University of Medical Sciences Ilam Iran; ^3^ Department of Microbiology, Faculty of Medicine Ilam University of Medical Sciences Ilam Iran

**Keywords:** chronic kidney disease, COVID‐19 mRNA vaccines, hemodialysis, SARS‐CoV‐2, the seroconversion rate, vaccine immunogenicity

## Abstract

**Background and Aims:**

Vaccine response is a concern in hemodialysis patients. Given that hemodialysis patients were not included in clinical trials, we aimed to synthesize the available evidence on the immunogenicity of coronavirus disease 2019 (COVID‐19) mRNA vaccines in hemodialysis patients.

**Methods:**

We searched Scopus, PubMed, Sciencedirect, and finally google scholar databases for studies on COVID‐19 mRNA‐vaccines immunogenicity in hemodialysis patients up to December 1, 2021. Eligible articles measured antibodies against severe acute respiratory syndrome coronavirus 2 (SARS‐CoV‐2) spike or Receptor‐Binding Domain Antibody (S/RBD) postimmunization with COVID‐19 mRNA vaccines. The immunogenicity of the vaccine was evaluated using seroconversion rates measured between 21 and 30 days after the first immunization and between 14 and 36 days post the second dose. We included studies including participants without a history of COVID‐19 before vaccination. Healthy controls or health‐care workers served as the control groups. After selecting eligible articles, the data were finally extracted from included articles. We used a random effects model to estimate the pooled seroconversion rate after COVID‐19 mRNA vaccine administration. We assessed the heterogeneity between studies with the *I* ^2^ statistical index.

**Result:**

We selected 39 eligible citations comprising 806 cases and 336 controls for the first dose and 6314 cases and 927 controls for the second dose for statistical analysis. After the first dose of mRNA vaccines, the seroconversion rate was 36% (95% confidence interval [CI]: 0.24–0.47) and 68% (95% CI: 0.45–0.91) in hemodialysis patients and the control group, respectively. While seroconversion rate after the second dose of mRNA vaccines was 86% (95% CI: 0.81–0.91) and 100% (95% CI: 1.00–1.00) in hemodialysis patients and the control group, respectively.

**Conclusion:**

Although the immune response of hemodialysis patients to the second dose of the SARS‐CoV‐2 mRNA vaccine is very promising, the seroconversion rate of dialysis patients is lower than healthy controls. Periodically assessment of antibody levels of hemodialysis patients at short intervals is recommended.

## 1 INTRODUCTION

Severe acute respiratory syndrome coronavirus 2 (SARS‐CoV‐2) was detected in late 2019 in Wuhan, China.^1^, ^2^ Coronavirus disease 2019 (COVID‐19) is one of the most critical health problems in the world right now.^3^, ^4^, ^5^ Several vaccines have been licensed for emergency use.^6^, ^7^ Vaccination remains an essential part of preventive care due to the high infection rate in hemodialysis patients. As kidney function decreases, the antibody response to the vaccine is impaired. Different methods have been used to improve response to influenza A and hepatitis B vaccines, such as higher vaccine doses and more frequent booster vaccinations.^8^ Vaccine efficacy in clinical trials has been determined for the general population. However, its efficacy has not been evaluated for vulnerable populations such as hemodialysis patients.

Hemodialysis patients are at high risk in the COVID‐19 pandemic due to increased average age, immunosuppression, renal failure, and frequent visits to dialysis centers. The mortality rate of COVID‐19 in these patients is much higher than in the general population, and up to 32% has been reported.^8^, ^9^ Including patients with kidney disease in the COVID‐19 vaccine, clinical trials are low. In most trials, people with “severe” or “chronic” kidney disease and people using/undergoing immunosuppression have been excluded. There is not much information about the effectiveness of COVID‐19 vaccines in hemodialysis patients. On the other hand, the effectiveness of previous vaccines such as hepatitis B and influenza in hemodialysis patients has been less than in the general population. The seroconversion rate after influenza virus vaccination is about 33%–80% in hemodialysis patients.^10^

The combination of this evidence has raised concerns about the vaccine's efficacy in this group and raised questions: The efficacy of COVID‐19 vaccines and the rate of postvaccination seroresponse in hemodialysis patients has not been fully determined; the duration of immune protection after vaccination is unknown. Seroconversion is related to immune protection from many pathogens, and there is increasing evidence that the same is true for SARS‐CoV‐2. Some studies have found a strong correlation between spike1 antibody titer and neutralization ability, innate immunity and the recruitment of T‐cell‐specific SARS‐CoV‐2 responses.^11^

Our objective was to synthesize the available evidence on the immunogenicity of COVID‐19 mRNA vaccines in hemodialysis patients compared with healthy controls.

## 2 METHODS

### 2.1 Systematic literature search

We conducted a systematic bibliographic search in the PubMed, Scopus, and Web of Science databases up to December 1, 2021, using the following keywords: (Severe acute respiratory syndrome coronavirus 2 or SARS‐CoV‐2) AND *(*Coronavirus Disease 2019 or COVID‐19*)* AND *(*vaccine efficacy) AND (hemodialysis or dialysis or kidney failure or chronic kidney disease) AND (seroresponse or antibody response or humoral response or serotiter or immunogenicity, effectiveness, or efficacy).

### 2.2 Eligibility criteria and study selection

Cohort and case–control studies published as of August 1, 2021, were searched. Other publications such as case reports, comments, conference abstracts, and review articles were excluded. We included all original studies on the efficacy or immunogenicity of COVID‐19 mRNA vaccines on hemodialysis patients. Studies written in languages other than English were excluded. Studies with participants without previous or active COVID‐19 infection met the inclusion criteria. In addition, the included studies measured immunoglobulin G (IgG) antibodies against SARS‐CoV‐ 2 S‐protein or RBD fragment. Vaccine immunogenicity was evaluated using seroconversion rates measured between 21 and 30 days after the first immunization and between 14 and 36 days post the second dose of COVID‐19 mRNA vaccines. Healthy controls or health‐care workers served as the control groups. The title and abstract of the articles were read. In the next step, the full text of the articles was evaluated for eligibility. After selecting the relevant studies, the reference list of each article was searched manually. Finally, all eligible articles were included in the study.

### 2.3 Data extraction

Two researchers independently recorded the following data: first author, year of study, country, type of study, number of cases, number of positive cases, number of the control group, number of positive controls, vaccine type, antibody type, timing post first/second dose (days). Discrepancies among the researchers were resolved through discussions or additional consultations with the third author.

### 2.4 Statistical analysis

The heterogeneity of studies was assessed using Cochran's *Q* test and the *I* ^2^ index. Due to the high heterogeneity, the random effect model was selected for meta‐analysis. Meta‐regression analysis was used to investigate the relationship between vaccine type and seroconversion rate. We used STATA version 11(STATA Corporation) for the analysis. All levels of significance tests were two‐sided and *p*‐values less than 0.05 was considered significant.

## 3 RESULTS

**Figure 1:**
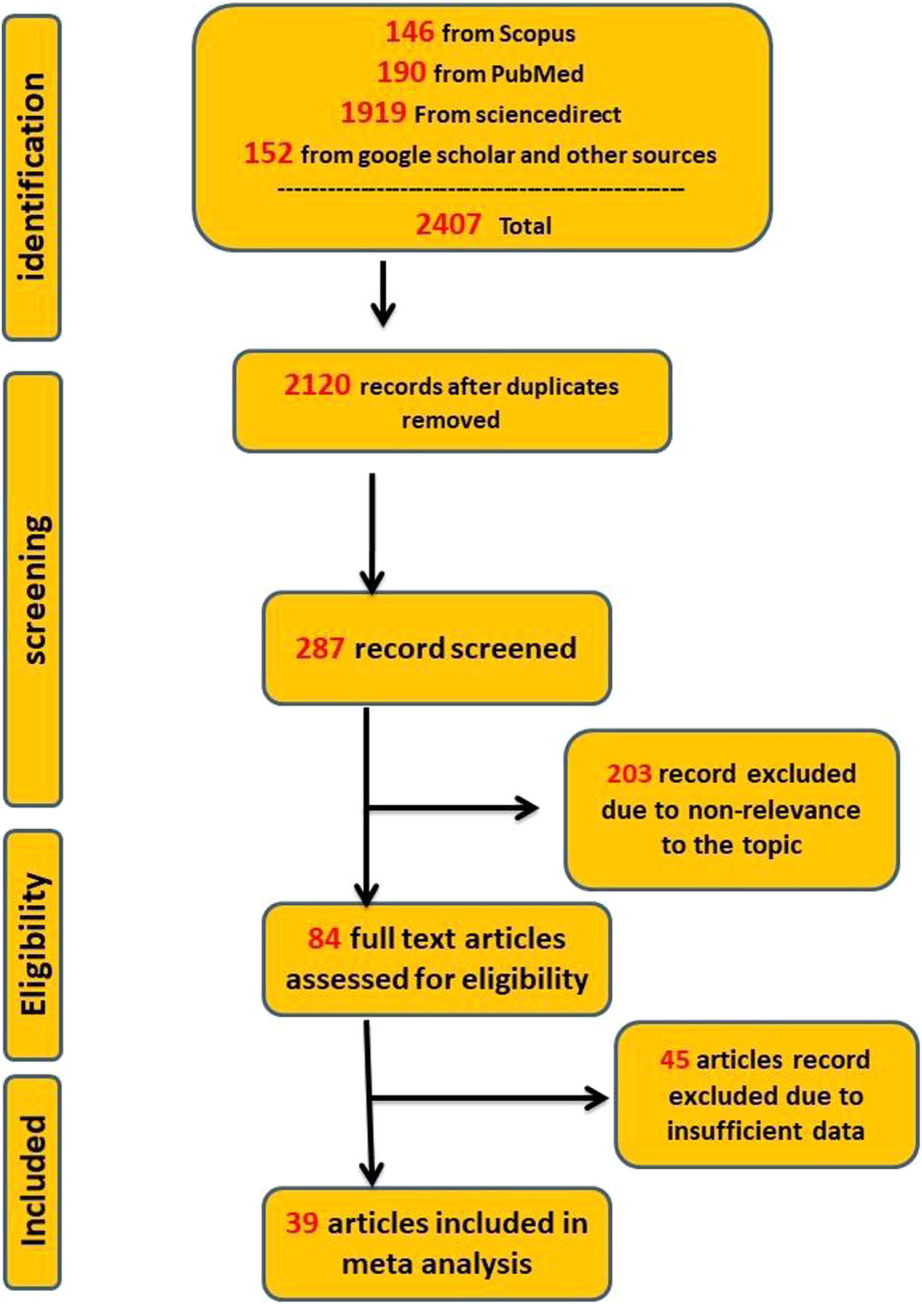
Flowchart of article identification and selection in the meta‐analysis

In the first research phase, a total of 2407 relevant articles were identified. After removing 2120 duplicate articles, 287 publications were reviewed for the title and abstract. Of these, 203 were removed due to irrelevance. The full text of another 84 articles was reviewed for eligibility criteria, and 45 articles were excluded due to insufficient data and no measurements of IgG antibody responses against S and RBD. Finally, 39 articles,^8^, ^12^, ^13^, ^14^, ^15^, ^16^, ^17^, ^18^, ^19^, ^20^, ^21^, ^22^, ^23^, ^24^, ^25^, ^26^, ^27^, ^28^, ^29^, ^30^, ^31^, ^32^, ^33^, ^34^, ^35^, ^36^, ^37^, ^38^, ^39^, ^40^, ^41^, ^42^, ^43^, ^44^, ^45^, ^46^, ^47^ including 806 cases and 336 controls for the first dose and 6314 cases and 927 controls for the second dose, for evaluating immunogenicity were included in the meta‐analysis. The flowchart of the article selection process is shown in Figure 1. Details of all included studies are provided in Tables 1 and 2.

**Table 1:** Antibody response data in dialysis patients after the first dose of mRNA vaccines

	(8, 12‐15)	Year	Country	Type of study	Number of cases	Number of positive cases (%)	Number of the control group	Number of positive controls (%)	Vaccine type	Antibody type	Timing post first dose (days)
1	Goupil et al.^12^	2021	Canada	Case–Control	131	56 (43%)	20	19 (95%)	BNT162b2	Anti‐RBD IgG	28
2	Torregiani et al.^13^	2021	France	Cohort	95	35 (36.84)	None	None	BNT162b2	Anti‐S IgG	21
3	Speer et al.^8^	2021	Germany	Case–Control	22	4 (18%)	46	43 (93%)	BNT162b2	Anti‐S1 IgG	17–22
4	Yau et al.^14^	2021	Canada	Case–Control	66	53 (80%)	35	None	BNT162b2	Anti‐S IgG	28
5	Attias et al.^15^	2021	France	Cohort	56	10 (18%)	None	None	BNT162b2	Anti‐S1 IgG	28
6	Longline et al.^16^	2021	France	Cohort	80	17 (21.5%)	None	None	BNT162b2	Anti‐RBD TOTAL	30
7	Weigert et al.^17^	2021	Portugal	Case–Control	143	42 (29.4%)	143	71 (49/7%)	BNT162b2	Anti S‐IgG	21
8	Broseta et al.^18^	2021	Spain	Cohort	78	44 (56.4%)	None	None	mRNA‐1273	Anti‐S IgG	28
9	Lesny et al.^19^	2021	Germany	Cohort	11	2 (18.18)	14	8	BNT162b2	Anti‐S IgG	2
10	Duarte et al.^20^	2021	Portugal	Cohort	42	21 (50%)	None	none	BNT162b2	Anti‐S IgG	21
11	Kolb et al.^21^	2021	Germany	Case–Control	32	3 (11%)	78	33 (42%)	BNT162b2	Anti‐RBD IgG	20
12	Zitt et al.^22^	2021	Austria	Cohort	50	21 (42%)	None	None	BNT162b2	Anti‐S IgG	28

**Table 2:** Antibody response data in dialysis patients after the second dose of mRNA vaccines

	Author	Year	Country	Type of study	Number of cases	Number of positive cases (%)	Number of the control group	Number of positive controls (%)	Vaccine type	Antibody type	Timing post second dose (days)
1	Grupper et al.^23^	2021	Israel	Case‐Control	56	54 (96%)	95	95 (100%)	BNT162b2	Anti–RBD IgG	30
2	Speer et al.^8^	2021	Germany	Case‐Control	17	14 (82%)	46	46 (100%)	BNT162b2	Anti‐S1 IgG	18–22
3	Frantzen et al.^24^	2021	France	Cohort	244	221 (91%)	None	None	BNT162b2	Anti‐S IgG	30
4	Simon et al.^25^	2021	Austria	Case‐Control	81	74 (91.35%)	80	80 (100%)	BNT162b2	Anti‐S total	21
5	Jahn et al.^26^	2021	Germany	Case‐Control	72	67 (93%)	16	16 (100%)	BNT162b2	Anti‐S IgG	14
6	Yau et al. ^14^	2021	Canada	Case‐Control	72	69 (96%)	35	35 (100%)	BNT162b2	Anti S‐IgG	14
7	Attias et al.^15^	2021	France	Cohort	52	43 (82%)	None	None	BNT162b2	Anti‐S1 IgG	21
8	Schrezenmeier et al.^27^	2021	Germany	Case‐Control	36	32 (88.9%)	44	40 (91%)	BNT162b2	Anti‐RBD IgG	21
9	Agur et al.^28^	2021	Israel	Prospective Cohort	122	114 (93.4%)	None	None	BNT162b2	Anti‐S IgG	36
10	Arevalo et al.^29^	2021	Germany	Case‐Control	44	31 (70/5%)	35	35 (100%)	BNT162b2	Anti‐S1 IgG	21–28
11	Longlune et al. ^16^	2021	France	Cohort	77	64 (83.11%)	NONE	NONE	BNT162b2	Anti‐RBD TOTAL	30
12	Strengert et al.^30^	2021	Germany	Case‐Control	81	95%	34	34 (100%)	BNT162b2	Anti‐S1 IgG	21
13	Lacson et al.^31^	2021	United States	Cohort	148	130 (88.1%)	None	None	BNT162b2	Anti‐RBD IgG	≥14
14	Broseta et al.^32^	2021	Spain	Cohort	75	69 (92%)	None	None	BNT162b2	Anti‐RBD IgG	21
15	Garcia et al.^33^	2021	United States	Cohort	416	369 (88.6)	None	None	BNT162b2	Anti‐RBD IgG	14–28
16	Ducloux et al.^34^	2021	France	Cohort	50	45 (90%)	None	None	BNT162b2	Anti‐RBD IgG	30
17	Garcia et al.^33^	2021	United States	Cohort	1316	1247 (94.8)	None	None	mRNA‐1273	Anti‐RBD IgG	14–28
18	Broseta et al.^32^	2021	Spain	Cohort	100	98 (98%)	None	None	mRNA‐1273	Anti‐RBD IgG	21
19	Lacson et al.^31^	2021	United States	Cohort	18	17 (94.4%)	None	None	mRNA‐1273	Anti‐RBD IgG	≥14
20	Espi et al.^18^	2021	France	Case‐Control	92	73 (79.35)	26	26 (100%)	BNT162b2	Anti‐RBD IgG	10–14
21	Espi et al.^32^	2021	France	Case‐Control	75	63 (84)	30	30 (100%)	BNT162b2	Anti‐RBD IgG	10–14
22	Weigert et al.^17^	2021	Portugal	Case‐Control	143	130 (90.9%)	143	136 (95.1%)	BNT162b2	Anti S‐IgG	21
23	Stumpf et al.^35^	2021	Germany	Cohort	936	908 (97%)	None	None	mRNA‐1273	Anti‐S1 IgG	28
24	Stumpf et al.^35^	2021	Germany	Cohort	200	176 (88%)	None	None	BNT162b2	Anti‐S1 IgG	35
25	Clarke et al.^36^	2021	England	Cohort	281	248 (88.3%)	None	None	BNT162b2	Anti S‐IgG	≥14
26	Dulovic et al.^37^	2021	Germany		76	72	23	23	BNT162b2	Anti‐RBD IgG	21
27	Hsu et al.^38^	2021	USA	Cohort	437	381	None	None	BNT162b2	Anti‐RBD IgG	14
28	Bertrand et al.^39^	2021	France	Cohort	45	8 (88.9%)	None	None	BNT162b2	Anti‐S1 IgG	14
29	Danthu et al.^40^	2021	France	Cohort	78	59 (85.5)	7	7	BNT162b2	Anti‐S IgG	14
30	Duarte et al.^20^	2021	Portugal	Cohort	42	6 (14%)	None	None	BNT162b2	Anti‐S IgG	21
31	Zitt et al.^22^	2021	Austria	Cohort	48	47(97.9%)	None	None	BNT162b2	Anti‐S IgG	14
32	Paal et al.^41^	2021	Germany	Cohort	179	173 (96.6)	70	68 (97.1)	BNT162b2	Anti‐S IgG	21
33	Giot et al.^42^	2021	France	Cohort	71	55 (77%)	None	None	BNT162b2	Anti‐S IgG	14
34	Dekervel et al.^43^	2021	France	Cohort	66	50	None	None	BNT162b2	Anti‐S IgG	14
35	Labriola et al.^44^	2021	Belgium	Case‐Control	24	19 (79%)	33	28 (85%)	BNT162b2	Anti‐RBD IgG	28
36	Kolb et al.^21^	2021	Germany	Cohort	32	28 (88%)	78	73	BNT162b2	Anti‐RBD IgG	14
37	Broseta et al.^18^	2021	Spain	Cohort	78	74 (94.9)	None	None	mRNA‐1273	Anti‐S IgG	21
38	Kaiser et al.^45^	2021	Austria	Cohort	77	76 (98.7)	None	None	mRNA‐1273	Anti‐RBD IgG	21
39	Kaiser et al.^45^	2021	Austria	Cohort	39	37 (94.8)	None	None	BNT162b2	Anti‐RBD IgG	21
40	Yanay et al.^46^	2021	Israel	Cohort	127	115 (90.5%)	132	132	BNT162b2	Anti‐S IgG	21–35
41	Tylicki et al.^47^	2021	Poland	Cohort	91	87 (95.6)	None	None	BNT162b2	Anti‐S IgG	14–21

**Figure 2:**
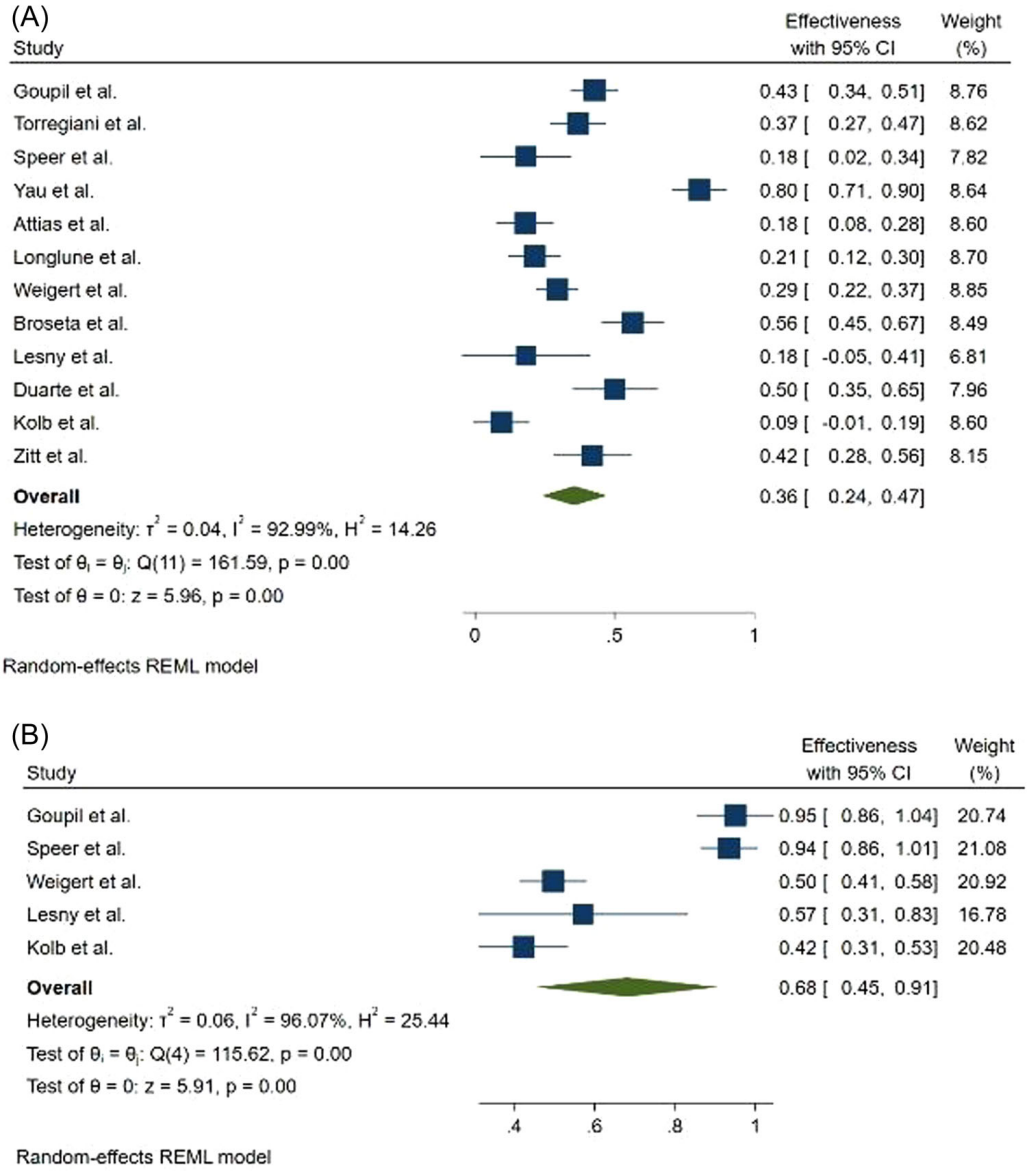
Forest plot for the seroconversion rate after the first dose of mRNA‐based vaccines (A) cases, (B) controls

**Figure 3:**
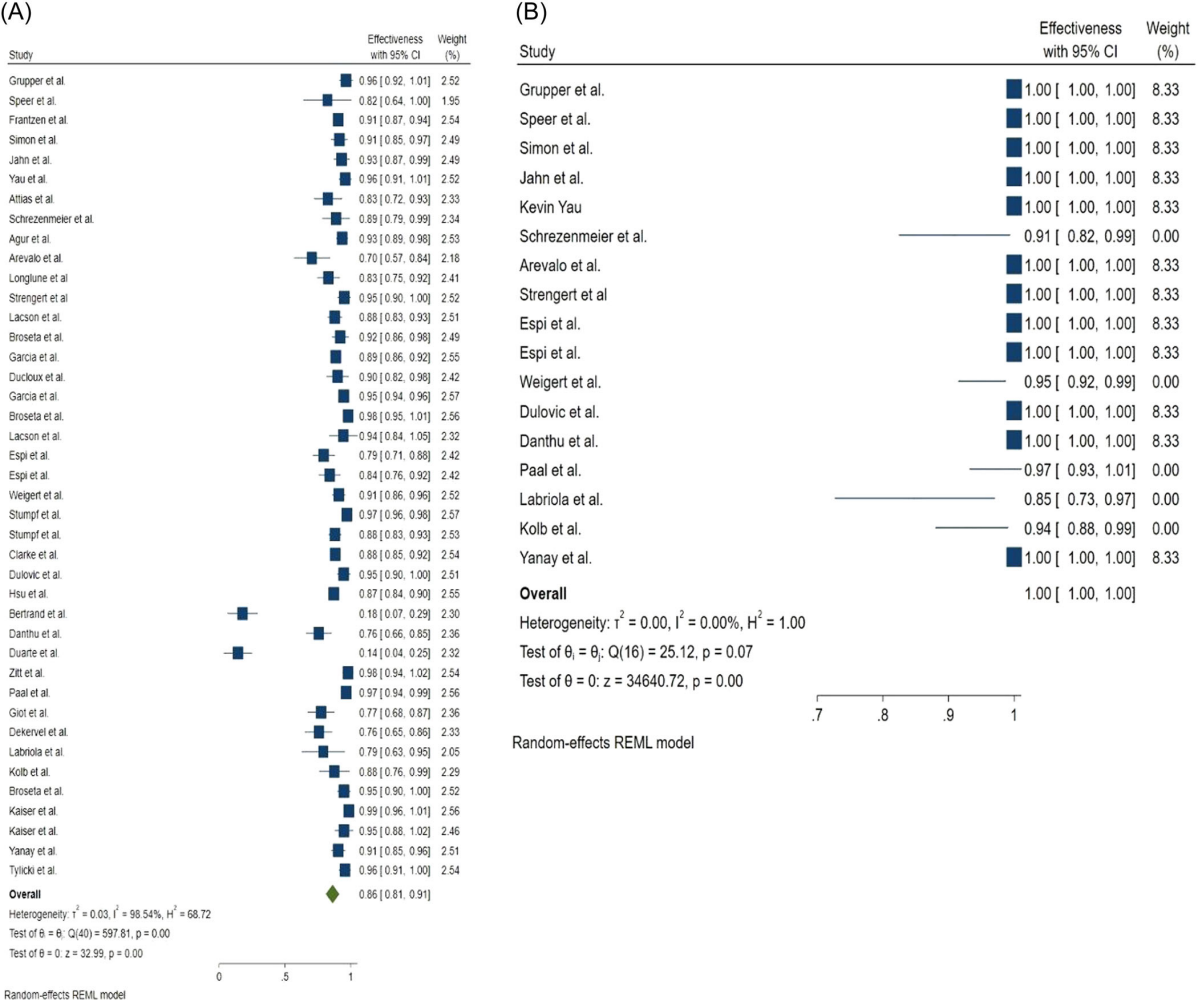
Forest plot for the seroconversion rate after the second dose of mRNA‐based vaccines, (A) Cases, (B) Controls

Seroconversion rate after the first dose of mRNA vaccines was 36% (95% CI: 0.24–0.47) and 68% (95% CI: 0.45–0.91) in hemodialysis patients and the control group, respectively (Figure 2). While seroconversion rate after the second dose of mRNA vaccines was 86% (95% CI: 0.81–0.91) and 100% (95% CI: 1.00–1.00) in hemodialysis patients and in the control group, respectively (Figure 3). Evaluation of the relationship between vaccine type and seroconversion rate using a meta‐regression model showed no significant differences between any of BNT162b2 and mRNA1273 vaccines and seroconversion rate after two doses of vaccine.

## 4 DISCUSSION

The mRNA‐based vaccines showed more than 90% efficacy in preventing COVID‐19 disease.^25^, ^48^ Patients with severe kidney disease were not present in clinical trials; therefore, the vaccine's efficacy in this high‐risk group has not been evaluated. Compared with the general population, hemodialysis patients have a lower response to hepatitis B and influenza vaccination.

Immunological changes include a distorted Th1/Th2 response, altered function of professional antigen‐presenting cells (APC), and susceptibility of B cells to apoptosis compared with people without kidney disease, making dialysis patients less likely to seroconvert and maintain protective serum titers over time.^31^, ^49^ After clinical trials and vaccine approval, several studies in dialysis centers examined seroconversion rates following the administration of two doses of mRNA‐based vaccines. The efficacy of vaccination was evaluated 14–30 days post the second injection by quantifying antibodies against spike protein, which shows a strong correlation with neutralizing antibodies.

Our results show that hemodialysis's seroconversion rate after the first dose of mRNA vaccines was 36% (95% CI: 0.24–0.47) and 68% (95% CI: 0.45–0.91) in patients and the control group, respectively. While seroconversion rate after the second dose of mRNA vaccines was 86% (95% CI: 0.81–0.91) and 100% (95% CI: 1.00–1.00) in hemodialysis patients and the control group, respectively.

The results of this meta‐analysis show that the seroconversion rate is low after the first dose of the vaccine, and the administration of the second dose should not be delayed. Although the seroconversion rate after the second dose in hemodialysis patients is lower than in the control group, it is very promising for hemodialysis patients.

Our results showed that mRNA‐based vaccines induce comparable seroconversion rates in hemodialysis patients and healthy controls. Our results indicate that the mRNA platform can be used to improve the immunogenicity of vaccines against other pathogens in hemodialysis patients.

The COVID‐19 mRNA‐based vaccine immunogenicity in hemodialysis patients is much greater than that of influenza and hepatitis B vaccines immunogenicity.

Several factors can contribute to the higher immunogenicity of mRNA‐based vaccines compared with the previous vaccines among hemodialysis patients. First, the vaccine platform is different; second, mRNA‐based vaccines’ efficacy has been studied in hemodialysis patients during the pandemic. It is possible that in some individuals, the combination of natural immunity after infection and vaccine‐generated immunity positively impacts the vaccine efficacy.^15^, ^50^ However, in some studies, initial/baseline infection or history of the previous infection of COVID‐19 has been actively monitored in the study population; these people were excluded from the study or located in a separate group.^15^, ^27^, ^28^, ^51^

The limitations of this study include: the small sample size, and also our study includes articles that assessed the efficacy of mRNA‐based vaccines by antibody titer. seroconversion and antibody titer is an easy method to evaluate the immunological response to vaccination, but it is not equivalent to complete protection.^52^ Also, the antibody levels required to protect against COVID‐19 have not yet been determined.^53^ However, to assess vaccine responses, it is recommended to assess both humoral and cellular responses.^54^ However, due to limited data on vaccine‐mediated cellular immunity, this study focused on investigating humoral immune responses after vaccination.

Although the seroconversion rate is high, many studies have reported that the antibody level of hemodialysis patients after being vaccinated with the COVID‐19 vaccine is lower than that of the control group; as a result, a shorter period of immune protection can be assumed. Therefore, it is necessary to periodically assess the antibody levels of hemodialysis patients at short intervals and renew their vaccination when necessary.

## 5 CONCLUSION

Although the immune response of hemodialysis patients to the second dose of SARS‐CoV‐2 mRNA vaccine is very promising, the seroconversion rate of dialysis patients is lower than healthy controls. As a result, it is necessary to pay more attention to the vaccination programs of this population, periodically assess the antibody levels of hemodialysis patients at short intervals and renew their vaccination when necessary.

## AUTHOR CONTRIBUTIONS

**Shahab Falahi**: Conceptualization; data curation; formal analysis; supervision; writing – original draft; writing – review & editing. **Hojjat Sayyadi**: Conceptualization; data curation; formal analysis; methodology; software. **Azra Kenarkoohi**: Conceptualization; data curation; formal analysis; project administration; supervision; writing – original draft; writing – review & editing.

## CONFLICT OF INTEREST

The authors declare no conflict of interest.

## TRANSPARENCY STATEMENT

The lead author Azra Kenarkoohi affirms that this manuscript is an honest, accurate, and transparent account of the study being reported; that no important aspects of the study have been omitted; and that any discrepancies from the study as planned (and, if relevant, registered) have been explained.

## Data Availability

Data sharing does not apply to this article as no datasets were generated or analyzed during the current study.
